# The effect of a GP’s perception of a patient request for antibiotics on antibiotic prescribing for respiratory tract infections: secondary analysis of a point-prevalence audit survey in 18 European countries

**DOI:** 10.3399/BJGPO.2024.0166

**Published:** 2025-04-24

**Authors:** Julie Domen, Rune Aabenhus, Anca Balan, Emily Bongard, Femke Böhmer, Valerija Bralić Lang, Pascale Bruno, Slawomir Chlabicz, Annelies Colliers, Ana García-Sangenís, Hrachuhi Ghazaryan, Anna Kowalczyk, Siri Jensen, Christos Lionis, Tycho M van der Linde, Lile Malania, Jozsef Pauer, Angela Tomacinschii, Akke Vellinga, Ihor Zastavnyy, Herman Goossens, Christopher C Butler, Alike W van der Velden, Samuel Coenen

**Affiliations:** 1 Department of Family Medicine & Population Health, Belgium, University of Antwerp, Antwerp; 2 Department of Public Health, University of Copenhagen, Copenhagen, Denmark; 3 BALAN MEDFAM SRL, Cluj Napoca, Romania; 4 Department of Primary Care Health Sciences, University of Oxford, Oxford, UK; 5 Institute of General Practice, Rostock University Medical Center, Rostock, Germany; 6 Department of Family Medicine, "Andrija Stampar" School of Public Health, School of Medicine, University of Zagreb, Zagreb, Croatia; 7 Département de Santé Publique, France, Université Côte d’Azur, Centre Hospitalier Universitaire de Nice, Nice; 8 Department of Family Medicine, Medical University of Bialystok, Bialystok, Poland; 9 Institut Universitari d’Investigació en Atenció Primària Jordi Gol (IDIAP Jordi Gol), Barcelona, Spain; 10 Wigmore clinic, Yerevan and Yerevan State Medical University, Armenia, Yerevan; 11 Centre for Family and Community Medicine, Faculty of Health Sciences, Medical University of Lodz, Lodz, Poland; 12 The Antibiotic Centre for Primary Care, Department of General Practice, University of Oslo, Oslo, Norway; 13 Clinic of Social and Family Medicine, School of Medicine, University of Crete, Heraklion, Greece; 14 Julius Center for Health Sciences and Primary Care, University Medical Center Utrecht, Netherlands, Utrecht; 15 National Center for Disease Control and Public Health, Tbilisi and Arner Science Management LLC, Tbilisi, Georgia; 16 DRC Drug Research Centre, Balatonfüred, Hungary; 17 University Clinic of Primary Medical Assistance of State University of Medicine and Pharmacy “N. Testemițanu”, Chişinǎu, Moldova; 18 School of Public Health, Physiotherapy and Sports Science, University College Dublin, Dublin, Ireland; 19 NGO “Academy of Family Medicine of Ukraine”, Lviv, Ukraine; 20 Laboratory of Medical Microbiology, Vaccine & Infectious Disease Institute, University of Antwerp, Antwerp, Belgium

**Keywords:** antibiotics, general practitioners, primary health care, respiratory tract infections

## Abstract

**Background:**

Illness severity, comorbidity, fever, age, and symptom duration influence antibiotic prescribing for respiratory tract infections (RTI). Non-medical determinants, such as patient expectations, also impact prescribing.

**Aim:**

To quantify the effect of a GP’s perception of a patient request for antibiotics on antibiotic prescribing for RTI and investigate effect modification by medical determinants and country.

**Design & setting:**

Prospective audit of general practices in 18 European countries.

**Method:**

Consultation data were registered of 4982 patients presenting with acute cough and/or sore throat. A mixed-effect logistic regression model analysed the effect of GPs’ perceptions of a patient request for antibiotics. Two-way interaction terms assessed effect modification. Relevant clinical findings were added to subgroups of lower RTI (LRTI), throat infection, and influenza-like-illness (ILI).

**Results:**

A GP’s perception of a request for antibiotics meant they were four times more likely to prescribe antibiotics (odds ratio [OR] 4.4, 95% confidence interval [CI] = 3.4 to 5.5). This effect varied by country: lower in Spain (OR 0.06), Ukraine (OR 0.15), and Greece (OR 0.22) compared with the lowest prescribing country. The effect was higher for ILI (OR 13.86, 95% CI = 5.5 to 35) and throat infection (OR 5.1, 95% CI = 3.1 to 8.4) than for LRTI (OR 2.9, 95% CI = 1.9 to 4.3). For ILI and LRTI, GPs were more likely to prescribe antibiotics with abnormal lung auscultation and/or increased or purulent sputum and for throat infection, with tonsillar exudate and/or swollen tonsils.

**Conclusion:**

GPs’ perceptions of an antibiotic request and specific clinical findings influence antibiotic prescribing. Incorporating exploration of patient expectations, point-of-care testing, and discussing watchful waiting into the decision-making process will benefit appropriate prescribing of antibiotics.

## How this fits in

GPs’ perception of a patient request for antibiotics is one of the strongest determinants of antibiotic prescribing, independent of medical determinants such as illness severity, comorbidity, fever, age, and symptom duration. The effect of a perceived patient request for antibiotics on prescribing varies by country. Specific clinical findings, abnormal long auscultation, increased or purulent sputum, tonsillar exudate, and swollen tonsils, also increase antibiotic prescribing. In patients for which the GP perceives a request for antibiotics, GPs could discuss a watchful waiting approach including safety netting and a follow-up visit.

## Introduction

Antibiotic resistant bacteria are one of the leading causes of death around the world with 4.95 million associated deaths in 2019, of which in 1.27 million a direct causal relationship was observed.^1^ Lower respiratory tract infections (LRTI) accounted for 1.5 million deaths associated with resistance, making them the most burdensome infections. Therefore, intervention strategies that reduce antibiotic resistance should be prioritised.

Important intervention strategies are minimising the use of antibiotics when they are not indicated and enhancing appropriate prescribing. Since most respiratory tract infections (RTI) are caused by viruses or are self-limiting anyway, antibiotics often provide no meaningful benefit.^2,3^ Specifically, for LRTI where pneumonia is not suspected, antibiotics do not significantly affect the duration of symptoms or the development of new or worsening symptoms.^4^ Similarly, the benefit of antibiotic treatment for throat infections is limited.^5^ Given the possible unnecessary side effects of antibiotics and their impact on antibiotic resistance, it is recommended to prescribe them only when appropriate according to guidelines.

About 80% of all antibiotics are prescribed in primary care.^6^ Our point-prevalence audit survey (PPAS) of GPs’ management of patients presenting with symptoms of acute cough or sore throat in general practices in 18 European countries showed that GPs are generally very confident about their decision to prescribe antibiotics.^7^ However, the considerable variation in antibiotic prescribing for these generally self-limiting condition indicates that this confidence is not always justified.^7^ Prior analyses on the PPAS data identified higher GPs’ perceived illness severity, longer illness duration, older age, fever, and presence of a comorbidity as independent determinants of antibiotic prescribing. Other studies showed that other than clinical findings, non-medical determinants, such as limited time and communication challenges, also influence antibiotic prescribing.^7–10^

Fischer *et al* studied the influence of medical determinants, patient symptoms, and physical findings, on antibiotic prescribing in primary care and found rales in lung auscultation, yellow sputum, pathologically altered tonsils, pathological cervical lymph nodes, fever, and fatigue to be associated with antibiotic prescribing.^11^ Coenen *et al* found that the effect of a perceived patient request on antibiotic prescribing depended on the clinical lung findings. GPs were more likely to prescribe antibiotics when they perceived a demand for antibiotics when the lung auscultation was normal, or with one abnormal auscultatory finding.^12^ In a study conducted in several European countries, the GP’s perception of patient demand was found to be the most important determinant of antibiotic prescribing for acute cough.^12^

Previous research indicates that GPs are often unconsciously influenced by their assumptions about their patients’ expectations, leading to unnecessary antibiotic prescribing.^13^ This phenomenon can be explained by the theory of planned behaviour (TPB), which suggests that antibiotic prescribing is influenced by behavioural intentions and the perceived control of prescribers.^14^ The TPB also includes a component of subjective norms, which measures the perceived social pressure a prescriber might experience.^14^ However, the independent effect of this perception on antibiotic prescribing and the interactions with medical and non-medical determinants have not yet been investigated, nor the effect in subgroups of patients diagnosed with LRTI, a throat infection, or ILI. Therefore, the purpose of the current study is to investigate which medical and non-medical determinants influence antibiotic prescribing, how these determinants modify the effect of a GP’s perception of a patient’s request for antibiotics, and to compare this effect between countries.

## Method

This is a secondary analysis of data collected in a PPAS of patient, clinical, and management characteristics of patients presenting with symptoms of acute cough or acute sore throat to primary care in 18 European countries that differed with respect to healthcare organisation, antibiotic use, and income level. GPs registered anonymous data from the consultation. Since no personally identifiable information was collected, patients were not asked to provide informed consent, which was approved by ethics committees in all participating countries.

### Setting

We recruited general practices from our European Primary Care Research Network in Armenia (*n* = 5), Belgium (*n* = 6), Croatia (*n* = 6), Denmark (*n* = 20), France (*n* = 18), Georgia (*n* = 5), Germany (*n* = 2), Greece (*n* = 6), Hungary (*n* = 5), Ireland (*n* = 6), Moldova (*n* = 4), The Netherlands (*n* = 12), Norway (*n* = 18), Poland (*n* = 8), Romania (*n* = 5), Spain (*n* = 6), Ukraine (*n* = 4), and the UK (*n* = 7). For each country, practices were asked to register approximately 240 patient consultations in January and February 2020.

### Inclusion characteristics

GPs were asked to sequentially register consultations with patients of all ages with symptoms of either acute cough (duration <28 days) and/or acute sore throat (duration <14 days), and to exclude patients with only nasal or ear symptoms.

### Data

Data could be entered directly during the consultation in Research Online, an online data-capture system, or entered later from paper case report forms (Appendix 1). Data fields covered the following: clinical presentation (patient characteristics, signs, and symptoms); GPs’ assessments (measurements of temperature, respiratory rate, heart rate, oxygen saturation, blood pressure); clinical examination findings; diagnostic testing (point-of-care; POC); patient (or parental) request for antibiotics (yes or no); the GP’s perception of illness severity (mild, moderate, severe; no guidance was provided for rating illness severity, therefore, rating was based on GPs’ own clinical insight); prescribing of antibiotics (yes or no). Unknown data for comorbidities, symptoms, and POC testing, which was <1%, were considered as not present or done. Missing data for most other variables, <1%, and <3% for duration of symptoms were not imputed (Table 1).

**Table 1.** A GP’s perception of patient request for antibiotics by country, age, infection subgroup, and comorbidity

	Yes (%) *n* = 611^a^ (12)	No (%) *n* = 4356^a^ (88)	*P* value^b^
**Country**			<0.0001
Armenia	34 (10)	321 (90)	
Belgium	16 (6)	272 (94)	
Croatia	11 (4)	259 (96)	
Denmark	23 (6)	366 (94)	
France	39 (15)	223 (85)	
Georgia	26 (11)	214 (89)	
Germany	5 (2)	235 (98)	
Greece	115 (49)*	118 (51)	
Hungary	28 (9)	273 (91)	
Ireland	79 (27)*	211 (73)	
Moldova	26 (11)	214 (89)	
Norway	16 (8)	195 (92)	
Poland	31 (13)	210 (87)	
Romania	20 (8)	227 (92)	
Spain	12 (4)	279 (96)	
The Netherlands	28 (9)	289 (91)	
Ukraine	59 (24)*	185 (76)	
UK	43 (14)	265 (86)	
**Age**			<0.001
0–19	147 (8)	1598 (92)	
20–49	221 (13)	1541 (87)	
50–100	241 (17)	1211 (83)	
**Comorbidities**			<0.0001
No	373 (11)	3159 (89)	
Yes	234 (17)	1183 (83)	
**Fever**			<0.0001
No	193 (9)	1951 (91)	
Yes	417 (15)	2399 (85)	
**Cough^c^**			0.21
No	97 (11)	771 (89)	
Yes	514 (13)	3575 (87)	
**Sore throat^c^**			0.003
No	215 (10)	1852 (90)	
Yes	394 (14)	2488 (86)	

Countries with the highest proportion of perceived antibiotic requests are highlighted in bold, with asterisk.^a^If numbers do not add up to the column total, this is owing to missing data. ^b^χ^2^ testing was used to compare variable proportions between patients for whom an antibiotic request was perceived and not. Two-sided testing at a 5% significance level were used. ^c^Cough and sore throat indicate a reported symptom, not a diagnosis

### Statistical analyses

Patient and management data are presented for the full sample. Categorical data are shown as absolute numbers and percentages, and continuous data as median with interquartile range (IQR: Q1–Q3) (Supplementary Table S1). The absolute numbers and percentages of patients from whom the GP perceived a request for antibiotics and those who did not request them are presented for each country, age category, presence of comorbidities, fever, cough, and sore throat. Differences between groups with and without perceived request were analysed with χ^2^ testing to compare proportions, using a two-sided 5% significance level (Table 1).

To investigate the effects of patient characteristics, symptoms, GPs’ perceptions of illness severity, GPs’ perceptions of an antibiotic request, and POC testing on antibiotic prescribing, we first performed univariable analyses using the Wald test. With a *P* value <0.05, the determinant was eligible for the multivariable model logistic regression model, which included a GP’s perception of a patient request for antibiotics as fixed effect. This new model was compared with the old model,^7^ only including country, illness severity, duration of symptoms, age, fever, and presence of any comorbidity as fixed effects and GP practice as random effect. Their goodness-of-fit was compared using the Akaike information criterion (AIC) of each model, with a lower AIC suggesting a better fit to the data.^15^

To investigate the contribution of the final determinants for the subgroups of LRTI, throat infection and ILI, univariable and multivariable analyses were repeated for: (1) patients with bronchiolitis, acute bronchitis, pneumonia, wheezing, and exacerbation of chronic obstructive pulmonary disease (LRTI); (2) patients with pharyngitis, tonsillitis, laryngitis, and peritonsillar abscess (throat infection); and (3) patients diagnosed with ILI. Subgroup-relevant clinical examination findings, such as dyspnoea, increased or purulent sputum, abnormal lung auscultation, chest pain, wheezing, and tachypnoea for LRTI; tonsillar exudate, swollen tonsils, tender cervical nodes, and peritonsillar abscess for throat infections; and all clinical examination findings for ILI, along with specific POC testing (C-reactive protein [CRP] test for LRTI and strep A for throat infection), were initially analysed through univariable analyses. Variables with a significance level of *P*<0.05 were included in multivariable models. The effect sizes of determinants are presented as odds ratios (ORs) with 95% confidence intervals (CIs) and *P* values. Consultations with other diagnoses available in the overall dataset, such as common colds, were not included in the subgroup analyses.

We examined interaction terms to determine whether the effect of a perceived antibiotic request on antibiotic prescribing is modified by patient characteristics (that is, age, comorbidities), illness characteristics (that is, duration of symptoms, presence of fever, perceived illness severity), POC testing, and country in the full sample, and for the clinical examination findings for the subgroups. To illustrate the effect of a perceived request for antibiotics across different countries, we present the predicted probability of antibiotic prescribing for a patient with selected characteristics with and without a perceived request for different countries. Statistical analyses were performed with R (version 4.3.1).

## Results

### Demographics

A total of 4982 consultations of patients presenting with symptoms of acute cough or sore throat were registered. Patient characteristics, clinical assessment, perception of illness severity, diagnosis, and management are shown in Supplementary Table S1. Most common diagnoses were ILI (22%), pharyngitis (21%), and bronchitis (17%). Only 3% of patients were classified as having severe disease and 2.3% were referred to hospital. The median age of all patients was 31 years (10–52 years). Of patients presenting with acute cough, 30% was prescribed an antibiotic; likewise, 31% of those presenting with acute sore throat. When fever was present, 35% of all patients were prescribed antibiotics and when at least one comorbidity was present, 44%. In patients with an illness severity perceived as mild, moderate, or severe, 15%, 49%, and 59% were prescribed antibiotics, respectively. The highest antibiotic prescribing was found in patients presenting to primary care in Ireland (54%), Hungary (52%), UK (44%), France (42%), Ukraine (42%), and Moldova (42%), while the lowest prescribing was found in Belgium (18%), Armenia (19%), Germany (19%), and Spain (19%). Overall, for 12% of patients a request for antibiotics was perceived, which was much higher in Greece (49%), Ireland (27%), and Ukraine (24%). Patients for whom a request for antibiotics was perceived were older, more likely to have comorbidities, presenting with a sore throat, or fever (Table 1).

### The independent effect of a perceived request for antibiotics on antibiotic prescribing

A multivariable logistic regression model, including the variables investigated before, now adding a GP’s perceived patient request for an antibiotic, showed that a perceived request exhibits a four-fold increase in the likelihood of receiving an antibiotic (OR 4.35, 95% CI = 3.41 to 5.54, Table 2). Including perceived request for antibiotics enhanced its goodness-of-fit (AIC = 4426) as compared with the previous model (AIC = 4575).^7^

**Table 2.** Multivariable analysis of determinants of antibiotic prescribing, taking into account a GP’s perception of a patient request for antibiotics (overall), and clinical examination findings for the subgroups diagnosed with a lower respiratory tract infection (LRTI), throat infection, and influenza-like-illness (ILI)

	All (*n* = 4746)		LRTI (*n* = 1519)		Throat infection (*n* = 1632)		ILI (*n* = 1046)	
	Adj OR^a^	95% CI	Adj OR^a^	95% CI	Adj OR^a^	95% CI	Adj OR^a^	95% CI
Use of POC test(s)	1.34	0.97 to 1.86	0.66	0.38 to 1.15	0.92	0.46 to 1.87	1.61	0.50 to 5.21
Illness severity: moderate	7.47*	6.22 to 8.96	5.38*	3.84 to 7.52	6.39*	4.35 to 9.31	4.65*	2.17 to 9.95
Illness severity: severe	9.94*	6.60 to 4.97	3.61*	1.99 to 6.53	1.46	0.39 to 5.45	3.27	0.46 to 23.28
Age (year)	1.01*	1.01 to 1.02	1.02*	1.01 to 1.03	1.01	1.00 to 1.02	0.99	0.97 to 1.01
Comorbidity	1.27*	1.04 to 1.55	1.05	0.75 to 1.48	1.08	0.67 to 1.72	1.37	0.60 to 3.13
Fever	1.50*	1.25 to 1.80	2.46*	1.77 to 3.42	1.18	0.67 to 1.73	1.59	0.69 to 3.66
Duration (day)	1.03*	1.01 to 1.04	1.02	1.00 to 1.05	1.06*	1.01 to 1.12	1.04	0.98 to 1.10
Request for antibiotics	4.35*	3.41 to 5.54	2.87*	1.92 to 4.31	5.05*	3.05 to 8.36	13.86*	5.47 to 35.11
Dyspnoea			1.35	0.96 to 1.90				
Chest pain			1.11	0.66 to 1.89				
Abnormal lung auscultation			3.09*	2.27 to 4.21			3.76*	1.51 to 9.37
Sputum			2.34*	1.73 to 3.16			2.65*	1.19 to 5.93
Wheeze			0.58*	0.41 to 0.82				
Tachypnoea			0.99	0.59 to 1.67				
Tonsillar exudate					16.43*	10.65 to 25.33		
Swollen tonsils					2.64*	1.84 to 3.80		
Tender cervical nodes					1.46	0.99 to 2.17		
Peritonsillar abscess					4.35	0.41 to 46.19		
Country	Appendix, Supplementary Table S2							

Multivariable mixed effects logistic regression for the dependent variable antibiotic prescribing controlling for age, comorbidity, fever, perceived illness severity, illness duration, perceived request for antibiotics, country and strep A and/or C-reactive protein (CRP) point-of-care (POC) testing for all patients. Three important subgroups, patients diagnosed with a LRTI, throat infection, or ILI were further studied; relevant clinical examination findings, with a significant association in univariable analysis, were included. CRP testing was included for patients with a LRTI and strep A for patients with a throat infection. Practice was included as random effect. No interaction terms were added to the models. Reference categories were ‘no’ for POC testing, comorbidity, fever,, and perceived request, ‘mild’ for illness severity, and Belgium (lowest antibiotic prescribing proportion) for country. For age and duration of symptoms an increase of one unit was considered. ^a^Adjusted odds ratios (OR) and 95% confidence intervals (CI); values significant at the 5% significance level in bold.

### The effect of a perceived request for antibiotics in patients diagnosed with LRTI, a throat infection, or ILI considering relevant clinical examination findings

Applying the new model to subgroups of patients diagnosed with LRTI, a throat infection, or ILI (Table 2), additionally adding clinical examination findings with significant association in univariable analysis — dyspnoea, chest pain, abnormal lung auscultation, increased or purulent sputum, wheezing, and tachypnoea for LRTI; tonsillar exudate, swollen tonsils, tender cervical nodes and peritonsillar abscess for a throat infection; and increased or purulent sputum and abnormal lung auscultation for ILI — shows that in patients diagnosed with a LRTI, abnormal lung auscultation (OR 3.09, 95% CI = 2.27 to 4.21) and increased or purulent sputum (OR 2.34, 95% CI = 1.73 to 3.16) increased the likelihood of receiving antibiotics, whereas patients with wheeze were less likely to receive antibiotics (OR 0.58, 95% CI = 0.41 to 0.82). In patients diagnosed with a throat infection, the presence of a tonsillar exudate significantly increased the likelihood of receiving antibiotics (OR 16.4, 95% CI = 10.65 to 25.33), and swollen tonsils were found to double the likelihood (OR 2.6, 95% CI = 1.84 to 3.80). In patients diagnosed with ILI, those with increased or purulent sputum were twice as likely (OR 2.65, 95% CI = 1.19 to 5.93) and those with abnormal lung auscultation three times as likely (OR 3.76, 95% CI = 1.51 to 9.37) to receive antibiotics. POC testing, fever, duration of symptoms, and perceived severe illness were no longer determinants of antibiotic prescribing in patients diagnosed with a throat infection and ILI. The effect of a perceived request decreased in patients with a LRTI (OR 2.87, 95% CI = 1.92 to 4.31), remained similar in those with a throat infection (OR 5.05, 95% CI = 3.05 to 8.36), and increased for patients with ILI (OR 13.86, 95% CI = 5.47 to 35.11). Supplementary Table S2 shows the likelihood of antibiotic prescribing in the complete study population for the different countries, taking perceived request into account.

### Country variation of the effect of a perceived antibiotic request on antibiotic prescribing

Table 1 shows that Greece (49%), the Ireland (27%), and Ukraine (24%) have the highest prevalence of perceived antibiotic requests. We investigated effect modification by country by incorporating an interaction term between country and perceived request, resulting in statistically significant interaction terms. The effect of a perceived request on antibiotic prescribing is significantly lower in Spain (OR 0.06, 95% CI = 0.01 to 0.52), Ukraine (OR 0.15, 95% CI = 0.04 to 0.66), and Greece (OR 0.22, 95% CI = 0.05 to 0.96) when compared with Belgium (also the reference in the previous analyses because of its lowest antibiotic prescribing proportion) (Supplementary Table S3). To put these data in context: GPs in those countries are less likely to prescribe antibiotics when they perceive a request for antibiotics than when a request is perceived in other countries. Consider a 50-year-old patient with symptoms of an RTI for 3 days, perceived by the GP as moderately severe ill. In Belgium, when the GP does not perceive a request, the predicted probability of antibiotic prescribing is 32%, while it is 82% when such a request is perceived. Conversely, in Greece, the corresponding probabilities are 39% and 59%, respectively. In Georgia and Poland, a non-significant higher influence of a perceived request was found. In all other countries, a perceived request for antibiotics has a similar impact on the probability of prescribing antibiotics for an RTI as in Belgium. Other determinants of antibiotic prescribing, clinical examination findings, patient’s age, comorbidity, fever, symptom duration, illness severity, and POC testing did not modify the effect of a perceived request on antibiotic prescribing (data not shown).

## Discussion

### Summary

This study confirms the effect of a GP’s perception of a patient’s request for antibiotics on their decision to prescribe antibiotics for RTI independently of other patient, clinical, and diagnostic determinants (fever, the GP’s perception of illness severity, age, comorbidity, duration of symptoms, POC testing, and signs and symptoms). Patients from whom GPs perceive a request for antibiotics were four times more likely to be prescribed an antibiotic. Moreover, this perceived request appeared to be the most important determinant of antibiotic prescribing next to the GP’s perception of illness severity. The effect of this perceived patient’s request on antibiotic prescribing varies between countries.

Additionally, we noticed a statisically significant influence of clinical examination findings on antibiotic prescribing. In patients with LRTI, antibiotic prescribing was more likely in patients with abnormal lung auscultation and increased or purulent sputum, and less likely in case of wheezing; in patients with ILI abnormal lung auscultation and increased or purulent sputum also increased the likelihood of antibiotic prescribing. In patients with a throat infection a tonsillar exudate and swollen tonsils increased the likelihood of antibiotic prescribing. When including clinical examination findings, strep A POC testing, fever, and illness severity no longer increased the likelihood of antibiotic prescribing in patients with a throat infection, while they did in an earlier model without this clinical information.^7^ The latter might be owing to the limited number of severe throat infections in the data, with a notable portion of such cases being referred to hospital immediately without antibiotics. This might also explain why in patients with LRTI the effect of severe illness on antibiotic prescribing is smaller than of moderately severe illness.

### Strengths and limitations

This study has several strengths making the findings highly valuable for understanding the multitude of interacting factors influencing antibiotic prescribing for RTI in primary care. First, the uniform and prospective data collection across multiple European countries enhances the applicability and comparability of findings within the European context, and enables relevant findings to be specifically linked to individual countries. Second, the use of an easy data-capture tool and the anonymous audit-type procedure for data collection ensured sequential patient registration; both factors contribute to a highly representative sample with minimal selection bias. Third, by considering the variety of determinants available during the consultation, our model gives a clear picture of how a perceived patient request for antibiotics influences the GP’s prescribing decision, taking into account all other determinants that interact in the decision-making process. Finally, the data enabled us to repeat the analyses for the subgroups of LTRI, throat infections, and ILI, indicating which determinants become more or less influential to antibiotic prescribing for these separate clinical indications; for example, fever only influences antibiotic prescribing for LRTI and not for throat infections and ILI.

A few limitations should be considered when interpreting the findings. First, although we conducted the study in multiple European countries, only GPs from 2–18 practices per country participated in the study, and the management policies of these practices may not be representative for their country. Therefore, conclusions based on between-country comparisons have to be taken with caution. Second, the impact of a GP’s perception of illness severity on antibiotic prescribing might be underestimated in this study, as severely ill patients are often directly referred to hospital. Third, it is important to note that GP-related factors influencing antibiotic prescribing decisions, such as external pressure, (diagnostic) uncertainty, limited time, previous experience, and communication challenges, were not included in our analysis.^9,10^

### Comparison with existing literature

The effect of a perceived patient request on antibiotic prescribing was explored and investigated before.^8,12,13,16–18^ Consistently, it has been identified as having a significant impact on antibiotic prescribing practice. The wording ‘perceived patient request’ arises from prior studies that have identified a mismatch between GPs’ perceptions of patient views and their patients’ actual views. Regarding actual patients’ views, there is also a distinction between patients’ expectations, hopes, implicit requests, and explicit requests for antibiotics.^8,16,19^ In the current study a ‘perceived patient request’ can mean both an implicit (perceived), and an explicit (directly communicated by the patient, or after the GP explored the patient’s expectations) wish for an antibiotic. This audit did not explore if antibiotics were discussed with the patient as part of shared decision making.

In our analyses, we thoroughly considered and evaluated this perceived request for antibiotics together with all available patient and clinical variables, including demographics, symptoms, perceived illness severity, clinical examination findings, POC testing, all of which contribute to a GP’s antibiotic prescribing decision. Despite this multitude of clinical determinants at hand during the consultation, a perceived request is a pivotal and persistent determinant for antibiotic prescribing. A systematic review exploring the impact of patient and prescriber characteristics, and clinical examination on antibiotic prescribing also identified age, fever, purulent sputum, tonsillar exudate, and illness severity to be associated with antibiotic prescribing. Interestingly, they found a stronger association with clinicians’ perception than with patients’ explicit expectations on antibiotic prescribing.^19^ This highlights the added value of explicitly exploring patients’ expectations during a shared decision-making process.

Interviews with GPs in Ireland suggested that high patient expectations for antibiotics are influenced by positive experiences.^20^ In addition, in Ireland 70% of the patients pay a high price to see their GP. In such cases, GPs’ clinical judgment may be compromised and inclined to meet this perceived expectation, leading to the unnecessary prescription of antibiotics. Research using the TPB confirms that behaviour is influenced by patients’ beliefs about positive consequences, highlighting the need to examine prescribing patterns carefully.^21,22^

### Implications for research and practice

The study identified pronounced between-country differences in a perceived request for antibiotics and the effect of this perception on antibiotic prescribing. We hypothesise that several contextual factors, patient, GP-related and organisationally, which were not captured in the audit, might account for these differences. Varying levels of patient antibiotic-related knowledge across countries has repeatedly been seen in the Eurobarometer studies.^23^ Such knowledge, together with acceptance of feeling ill for a few days, health and healthcare perception, will influence patients’ requests. In addition, available time for a consultation, the doctor–patient relationship, national background levels of antibiotic prescribing, and antimicrobial resistance stewardship programmes might affect how GPs handle a perceived request. How these factors interfere and influence antibiotic prescribing needs to be further explored for the benefit of targeted intervention strategies.

The disappearing effect of strep A POC testing when incorporating clinical examination findings into the model of antibiotic prescribing for throat infections, suggests that after clinical examination, the decision to prescribe antibiotics remains consistent irrespective of the strep A test results, and that GPs rely more on their clinical judgment than on the result of a strep A test. As POC testing was only performed in 11% of the consultations more data on management of throat infections are needed to interpret the added value of POC testing in different relevant scenarios; for example, the influence of a negative strep A test result when a tonsillar exudate is present. Additionally, by incorporating clinical findings into the subgroup models, we observed that the effect of a perceived request remains approximately the same for throat infections. However, the effect of a perceived request seemed to decrease for LRTI when clinical findings were taken into account, from an OR of 4.36 (95% CI = 3.43 to 5.56) to 2.87 (95% CI = 1.92 to 4.31). This difference is not statistically significant and may therefore be attributed to chance. Nevertheless, it suggests that GPs are less influenced by a perceived request in LRTI than in throat infections, maybe because in LRTI they have greater confidence in the value of clinical findings.

Given the significant impact of GPs’ perception of a patient request for antibiotics, which often outweighs actual patient wishes, it is crucial to prioritise interventions that close the communication gap between patients and GPs. Although GPs may be reluctant to explicitly ask about patient expectations to avoid potential discussions, incorporating expectation exploration concerning the management of RTI into a shared decision-making process is a key strategy to reduce unnecessary antibiotic prescribing.^19^

The clinical findings fever, abnormal lung auscultation, and/or increased or purulent sputum with LRTI and ILI, and tonsillar exudate or swelling with throat infections increase antibiotic prescribing. Therefore, additional consideration is needed for patients presenting with those clinical findings. POC or laboratory-based testing could help decreasing diagnostic uncertainty to more appropriately target antibiotic treatment. Additionally, safety-netting advice, information leaflets, and/or planning a follow-up consultation (in-person or remote) could aid in a more cautious approach to antibiotic prescribing. Finally, GP education, showing the strong association between tonsillar exudate and swelling with antibiotic prescribing without clear benefits of antibiotics for throat infections, could help reducing unnecessary prescribing for this condition.^5,24^

In conclusion, our study underscores the importance of a GP’s perception of a patient request for antibiotics in the multifactorial nature of GPs’ antibiotic prescribing decisions for RTIs, involving both medical and non-medical determinants. We found that a GP’s perception of illness severity, perceived patient request for antibiotics, and several clinical examination findings significantly and independently influence antibiotic prescribing. For antibiotic stewardship in primary care, these findings highlight the value of communication skills to minimise the gap between a perceived patient request and real patient needs, diagnostic testing in patients with specific clinical findings, and shared decision making, with safety netting towards a watchful approach of managing patients with RTI.

## Data

The dataset relied on in this article is available from the corresponding author on reasonable request.
